# The suppression of FOXM1 and its targets in breast cancer xenograft tumors by siRNA

**DOI:** 10.18632/oncotarget.359

**Published:** 2011-12-25

**Authors:** Ming Wang, Andrei L. Gartel

**Affiliations:** ^1^ Department of Medicine, University of Illinois at Chicago, Chicago, IL, 60612, USA; ^2^ Department of Biochemistry and Molecular Genetics, University of Illinois at Chicago, Chicago, IL, 60607, USA; ^3^ Department of Microbiology and Immunology, University of Illinois at Chicago, Chicago, IL, 60612, USA

**Keywords:** breast cancer, xenograft tumors, FOXM1, siRNA

## Abstract

As an oncogenic transcription factor, the Forkhead box protein M1 (FOXM1) is overexpressed in human tumors. FOXM1 promotes tumorigenesis by regulating genes associated with cell cycle progression and cell proliferation, and its inhibition in cell lines has been shown to sensitize cells to apoptosis. In this report, we examined the possibility of suppressing FOXM1 in tumors *in vivo*, through the administration of FoxM1-specific siRNA. Firstly, we determined the functionality of siRNA treatment in subcutaneous MDA-MB-231-luc breast cancer tumors. We found that upon encapsulation into a PEI-based delivery agent, fluorescently-labeled siRNA was retained within tumors when administered intratumorally. Injection of anti-luciferase siRNA was also able to suppress tumor-associated luciferase for at least 48 hours. More importantly, repeat administrations of PEI-encapsulated anti-FoxM1 siRNA resulted in the reduced expression of FOXM1 protein levels in tumors. In addition, both the protein levels and mRNA levels of cdc25B and Aurora B Kinase, transcriptional targets of FOXM1 were also reduced in tumors treated with anti-FoxM1 siRNA. p27, an indirect target of FOXM1 associated with growth inhibition was further found be increased in tumors treated with FoxM1-siRNA. Our data suggests that anti-FoxM1 siRNA can be functional when administered into tumors in an *in vivo* system, and that anti-FoxM1 siRNA holds potential as part of a therapy for cancer treatment.

## INTRODUCTION

The Forkhead box protein M1, FOXM1, serves as a transcription factor for a wide range of genes relating to cell proliferation, cell cycle progression, adult tissue homeostasis, repair of DNA damage, and angiogenesis [1-4]. FOXM1 is also considered to be an oncogenic transcription factor, as its expression in cancer cells is found to be abnormally high [5-10]. In fact, genomic studies have identified FoxM1 to be one of the highest expressed genes in a wide range of human tumors [5, 11, 12], where a correlation between tumor aggressiveness and FOXM1 expression levels have been shown [8, 9, 13]. FOXM1's role in tumorigensis is based on its regulation of cell cycle progression, particularly the G1/S and G2/M transition and M phase progression. For example, FOXM1 has been found to regulate cell cycle-associated genes such as the centrosome proteins CENPA, CENPB and CENPF, Cdc25B, cyclin B, Aurora B kinase, survivin, and polo-like kinase 1 (PLK1) [7, 14-19]. Consequently, several of such genes are also overexpressed in cancers, and contribute to the progression of cancer development. Furthermore, FOXM1 also negatively regulates the expressions of cyclin-dependent kinase inhibitors p21 and p27, through transcription regulation of Skp2 and Cks1 [3, 20]. In addiiton, *in vitro* studies have shown that the suppression of FOXM1 by siRNA sensitizes cancer cells to cell death upon stimulation with conventional chemotherapeutic drugs [21-23].

Currently, the implementation of RNAi for *in vivo* purposes is challenging, especially in the development of nanoparticle carriers for the transportation of siRNA to tumors. Examples of successful delivery of siRNAs to tumors are widely documented in literature, although few have been developed to the stage of clinical applicability [24-26]. Recently, demonstrated is the engineering of a cyclodextrin-based nanoparticle system, which was shown to successfully aid the delivery of anti-RRM2 (M2 subunit of ribonucleotide reductase) siRNA to melanoma cancers in human patients [27]. As research in tumor-targeted siRNA delivery steadily advances, we examine the functional ability of anti-FoxM1 siRNA to induce suppression of FOXM1 in tumors, as a proof-of-principle for the potential of anti-FoxM1 siRNA as a therapeutic agent.

## RESULTS AND DISCUSSION

### PEI-encapsulated siRNA is retained in tumor xenografts for a minimum of 24 hrs upon intratumoral administration

Here, we chose a polyethylimine-based cationic poymer, JetPEI (Polyplus) as an encapsulation agent for the *in vivo* delivery agent for siRNA. JetPEI was complexed at an N-to-P ratio of 8, with the siRNAs: anti-FoxM1-siRNA, control siRNA and control-FITC siRNA. N refers to the number of positively charged amine groups in PEI and P represents the number of negatively charged phosphates in the siRNA backbone. Due to the nature of PEI-siRNA complexes (overall cationic in zetapotential, no protective ‘stealth’ layer), intravenous injection would not result in its accumulation into subcutaneous tumors [28-30]. The only injection method for successful delivery to such tumors would be direct intratumoral injection.

**Figure 1:**
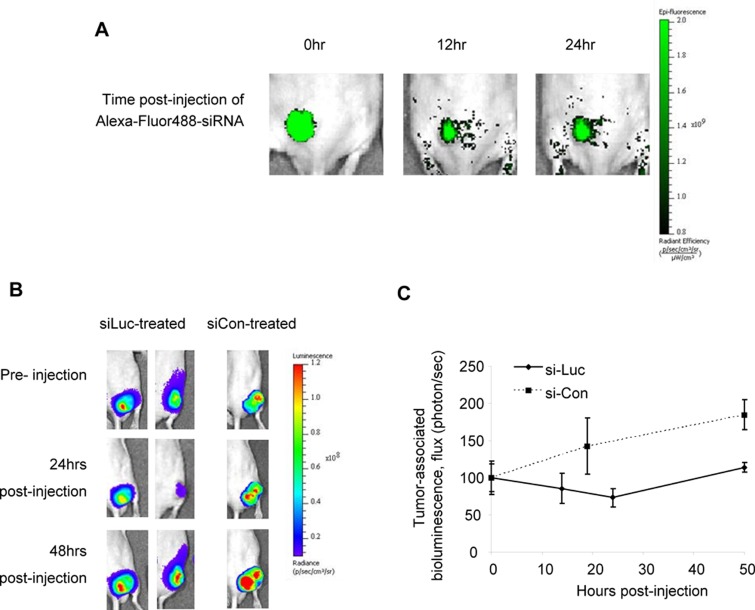
PEI-encapsulated siRNA is retained in tumors and is functional for protein suppression A, Fluorescently-labeled siRNA, when encapsulated into JetPEI delivery agents, can be retained in subcutaneous xenograft tumors upon intratumoral delivery for at least 24 hours, as determined by the persistence of tumor-associated fluorescence. B, Suppression of tumor-associated luciferase was apparent 24 hours post-injection of luciferase-specific siRNA (10μg) and was maintained for at least 48 hours. Tumor-associated luciferase continued to increase in control siRNA-treated tumors. C, Quantification of flux (photons/sec) in tumors treated with luciferase-specific and control siRNA. *n*=2−4 tumors, where values depict averages and error bars represent SD.

PEI-encapsulated Fluorescent-siRNA (control sequence) was first used to visualize the retention of siRNA within subcutaneous xenograft tumors upon intratumoral delivery. MDA-MB-231-luc xenografts were prepared in nude mice, after which 10μg of siRNA (encapsulated with 1.6μL of JetPEI) was administered to tumors intratumorally. The siRNA-associated fluorescence in the mice was monitored by whole-body live fluorescence imaging, using an excitation wavelength of λ=465nm and an emission wavelength of λ=550nm. We found that PEI-encapsulated siRNA was retained within tumors for at least 24 hours, as determined by the retention of tumor-associated fluorescence over time (Figure 1, A). This demonstrates that PEI-encapsulated siRNA can be retained in tumors and potentially can induce its functional effect upon intratumoral injection.

Presently, methods for the functional delivery of siRNA to tumor sites as a means for cancer therapy remain limited. The phosphate backbone renders siRNA to be negatively charged, a nature that prevents its cellular internalization, through electrostatic repulsion with negatively charged cell membranes [31]. In addition, siRNA is vulnerable to digestion by RNAase enzymes, if administered to biological systems without an exterior carrier [28, 29]. Required, therefore, are carrier systems that can transport siRNA to tumor sites before its action on protein suppression can be realized. The development of nanomaterials for such purposes has attracted considerable attention and since, the successful delivery of siRNA to melanoma cancers by intravenous injection has been shown in humans [27]. As the delivery of siRNA to tumors becomes a clinical reality, researchers have relied on adopting the method of intratumoral injection to examine the feasibility of suppressing specific proteins as part of a chemotherapeutic treatment [29, 30, 32, 33].

### PEI-encapsulated anti-luciferase siRNA inhibits luciferase expression in luciferase-expressing tumors

To further elucidate the functional suppression of siRNA-specific proteins in such tumors, a non-evasive method involving MDA-MB-231-luc (luciferase-expressing) xenograft tumors and anti-luciferase siRNA (siLuc) was firstly adopted. PEI-complexed anti-Luciferase siRNA was injected intratumorally into luciferase-expressing tumors and the tumor-associated-luciferase expression was monitored by bioluminescence imaging (Figure 1, B, C). It was found that luciferase suppression by anti-luciferase siRNA was apparent 24 hours after injection, followed by a slow recovery of luciferase levels to that of control-treated tumors (Figure 1, B, C). Administration of control siRNA (siCon) had no effect on luciferase expression in MDA-MB-231-luc tumors, where the bioluminescence increased steadily overtime. Here, we show that the JetPEI-siRNA formulation is capable of inducing protein-specific suppression in tumors, and is therefore useful for the administration of therapeutic siRNAs. It should be considered that the duration of protein suppression depends on both the half-life of the targeted protein plus the proliferation rate of the cells; however, the time frame of 24-48hrs provides a guideline for intervals in a dosing schedule for repeat siRNA administration. It is acknowledged that intratumoral injection of siRNA is not the preferred method for *in vivo* RNAi experimentation, but here it is unavoidable until tumor-targeted systemic administrations of such drugs are fully developed.

### In vitro suppression of FOXM1 and its targets in MDA-MB-231-luc cells by anti-FoxM1 siRNA

**Figure 2:**
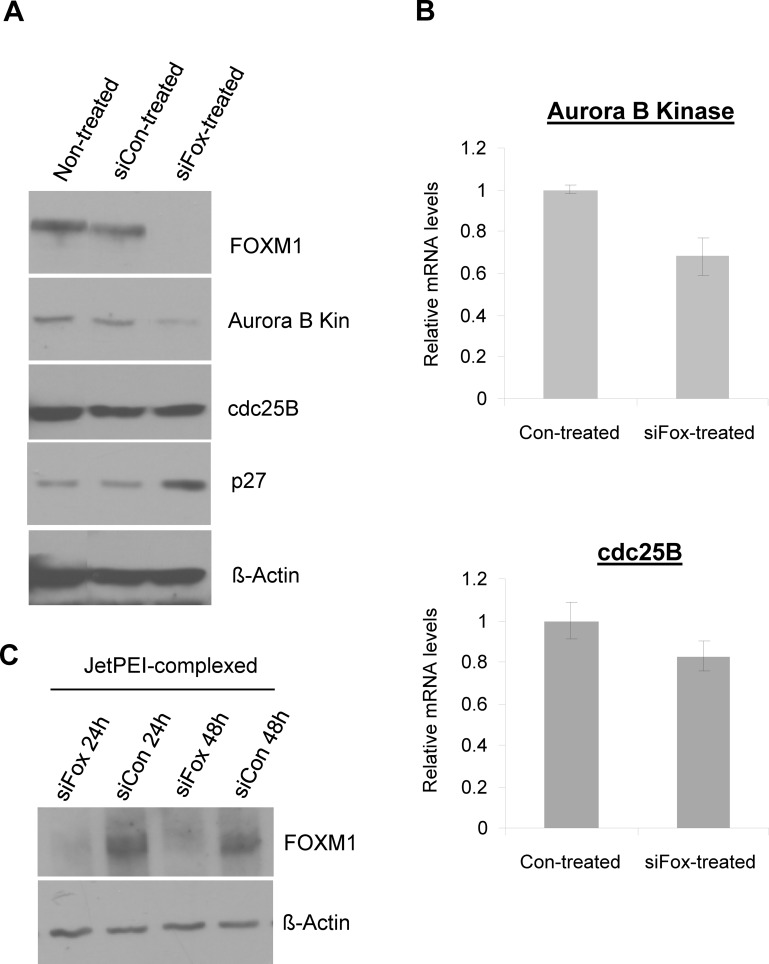
FoxM1-specific siRNA (siFox) suppresses the expression of FOXM1 and its targets in MDA-MB-231-luc cells A, siFox (50nM, delivered *via* Lipofectamine2000, 48 hours post-transfection) suppresses the protein levels of FOXM1 and its direct transcriptional targets Aurora B Kinase and cdc25B, and elevates levels of an indirect target of p27. B, siFox (50nM) inhibits the mRNA levels of Aurora B Kinase and cdc25B. *n*=3, values depict averages and error bars represent SD. C, JetPEI-encapsulation of siFox (50nM) is also functional in suppression FOXM1 and is therefore feasible as a delivery agent to tumors.

The effects of anti-FoxM1 siRNA (siFox) on the expression of FOXM1 and FOXM1's targets were examined in MDA-MB-231-luc cells *in vitro*. In conjunction with FOXM1 suppression, anti-FoxM1 siRNA was also able to inhibit the expression of FOXM1 transcriptional targets (Figure 2, A, B). Specifically, the protein levels of Aurora B Kinase and cdc25B were found to be suppressed after a 48 hr treatment with anti-FoxM1 siRNA (Figure 2, A). Additionally, reductions in mRNA levels of FOXM1's transcriptional targets were also observed, upon transfection with anti-FoxM1 siRNA (Figure 2, B). As targets of FOXM1 are also associated with cell proliferation, they too are being pursued as potential targets for cancer treatment. Examples include drugs such as HQPA (hydroxyquinazoline pyrazol anilide) and NSC663284 [6-chloro-7-(2-morpholin-4-ylethylamino)quinoline-5,8-dione], small molecules used for the inhibition Aurora B Kinase and cdc25B, respectively [34-38]. In this case, the effect of FOXM1 suppression is likely similar to the effect of inhibiting individual FOXM1 targets, as suppression of proteins further upstream of molecular pathways may lead to the suppression of a range of targets that contribute to cancer cell viability. Additionally, p27, a non-direct, secondary target of FOXM1 was found to be up-regulated by FOXM1 suppression, because of the down-regulation of Skp2, a component of the SCF-Skp2 ubiquintin ligase that targets 27 for proteolytic degradation (Figure 2, A) [3, 20]. This suggests that the reduction of the protein levels of FOXM1's targets is through suppression of their mRNA transcription.

We further demonstrated the applicability of JetPEI to act as a delivery agent of siRNA, shown by the induced suppression of FOXM1 in MDA-MB-231 cells upon treatment with JetPEI-complexed anti-FoxM1 siRNA (Figure 2, C). The inhibition of FOXM1 protein expression by JetPEI-siRNA was maintained for at least 48hrs, whilst treatment with controls (JetPEI complexed with non-functional control siRNA, siCon) had no effect on FOXM1 expression (Figure 2, C).

FOXM1, with its roles in cell proliferation, along with its overexpression cancer cells, is becoming increasingly highlighted as a drug target for cancer therapy. Molecules such as ARF-peptide [39], thiazole antibiotics thiostrepton and Siomycin A [40-42], proteasome inhibitors in general [43] and other small molecules [44] have been shown to be suppressors of FOXM1 *in vitro* and *in vivo*. However, such drugs, particularly the proteasome inhibitors, are likely to operate through multiple mechanisms that may affect numerous proteins, not just that of FOXM1 [43, 44]. On the other hand, siRNA is known to selectively inhibit target proteins, therefore inhibition of FoxM1 by anti-FoxM1 siRNA will be specific way to target FOXM1.

### Repeat intratumoral injection of PEI-encapsulated anti-FoxM1 siRNA suppresses the expression of FOXM1 and its targets in MDA-MB-231-luc tumor xenografts

**Figure 3:**
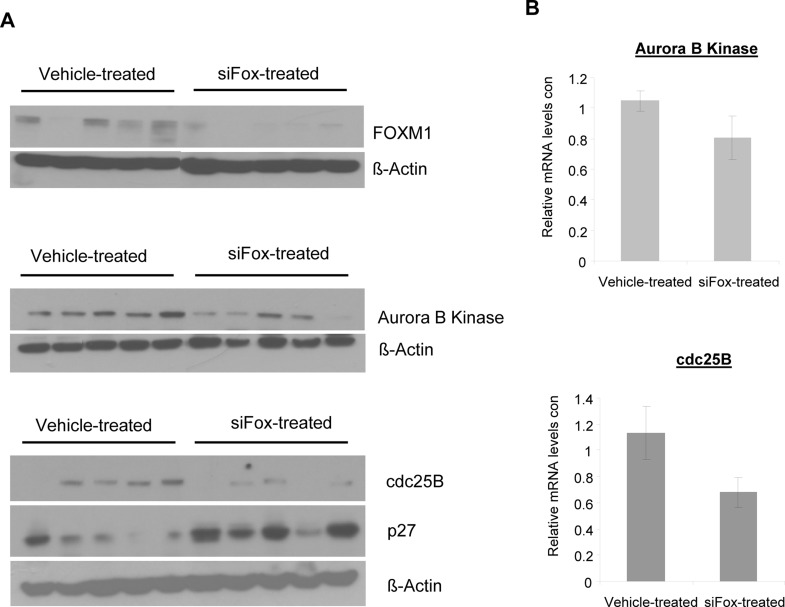
Repeat injection of siFox reduces protein expressions of FOXM1 and its targets in MDA-MB-231-luc subcutaneous tumors A, After 10 intratumoral injections of siFox (10μg, complexed with 1.6μL JetPEI, once every two days, over 20 days), protein levels of FOXM1, Aurora B Kinase and cdc25B were suppressed, compared to tumors treated with JetPEI-only controls. Western blots represent 5 individual tumors from each treatment group. B, siFox also reduced the mRNA levels of FOXM1 transcriptional targets, as demonstrated by quantitative PCR. *n = 5−8* where values depict averages and error bars represent SEM.

MDA-MB-231-luc cells were implanted subcutaneously into the flanks of nude mice and allowed to proliferate until tumors reached sizes of ~200mm^3^. JetPEI-encapsulated anti-FoxM1 siRNA was prepared and administered intratumorally to tumors at a dose of 10μg siRNA/tumor, once every other day (3-4 times per week). Control tumors received intratumoral injections of JetPEI only, as previous experiments showed that control siRNA had no effect on protein expression (Figure 1, B and Figure 2, A). After 10 injections (20 days), tumors were removed and analyzed for protein and mRNA levels of FOXM1, Aurora B Kinase, cdc25B and appropriate controls. We found that the protein levels of FOXM1 were effectively reduced in tumors treated with repeat administration of siFox siRNA, compared to that of control-treated tumors (Figure 3, A). Complying with *in vitro* results, protein levels of Aurora B Kinase (Figure 3, A) and cdc2B (Figure 3, A) were also found to be suppressed in tumors treated with siFox siRNA. Again, the mRNA levels of the FoxM1 targets were consistently reduced in tumors subjected to siFox injections (Figure 3, B). A reduction in FOXM1, Aurora B Kinase and cdc25B also corresponded to an increase in levels of p27, the cyclin-dependent kinase inhibitor that is indirectly negatively regulated by FOXM1 (Figure 3, A). These findings suggest that the injection of PEI-encapsulated siRNA is functional in such a tumor model, and that FOXM1 suppression also leads to the repression of its transcriptional targets.

As FOXM1 is known as a master regulator of the cell cycle, its suppression inhibits the transcription of genes associated with proliferation and tumor growth, and therefore may be beneficial to part of a cancer treatment. In conjunction with *in vitro* studies, suppression of FOXM1 in tumors by siRNA also realized the suppression of its targets. As with our *in vivo* data, other studies have also described the depletion of FOXM1 targets such as cdc25B and Aurora B Kinase in FOXM1-null cells [3, 15]. The suppression of FOXM1 and its targets also corresponds to the upregulation of p27, a Cdk inhibitor protein that is negatively regulated on protein level by the FOXM1 target, Skp2 [39]. Particularly, high FOXM1 expression has been found to increase resistance towards certain anticancer drugs [21-23], and its inhibition may sensitize cancer cells towards current chemotherapeutic drugs. If the suppression of FOXM1 by siRNA is feasible and efficient in mouse tumor models, it may pave way for the development of RNAi-based therapies for FOXM1-targeting in human tumors.

## METHODS

### Materials

MDA-MB-231-luc-D3H2-LN, human lymph node-derived metastatic mammary gland adenocarcinoma (Caliper Lifescience) were maintained in MEM media (Mediatech) supplemented with 10% FBS (Atlanta Biological), 1% 100X non-essential amino acids (Gibco), 1% 200mM NaPyruvate (Gibco) and 75μg/mL Zeocin (Invitrogen). JetPEI and 20% glucose solution was obtained from Polyplus, Lipofectamine 2000 was purchased from Invitrogen and Optimem was purchased from Gibco. Trizol was obtained from Ambion, High capacity cDNA Reverse Transcription kit was purchased from Applied Biosystems and SYBR Green fast start universal was obtained from Roche. All primers were synthesized by Integrated DNA Technologies. Anti-FoxM1 and control siRNAs were purchased from Sigma, Fluorescent-siRNA (Allstars Negative Alexa Fluor 488) and anti-Luciferase GL3 siRNA were purchased from Qiagen. D-Luciferin (potassium salt) was obtained from Gold Biotechnology.

## METHODS

### Treatment of cells in vitro

MDA-MB-231 cells were seeded at 5x10^4^ cells/3cm plate in antibiotic-free media and incubated overnight before treatment. siRNA was complexed with either Lipofectamine2000 or with JetPEI before administration to cells. For complexation with Lipofectamine2000: in one vial, 1.5μL siRNA (100μM in nuclease-free H_2_O) was diluted in Optimem (50μL), and in another vial, 2.25μL of Lipofectamine2000 was diluted in Optimem (50μL). The Lipofectamine2000 solution was added to the siRNA solution and mixed by pipetting. The siRNA/liposome complexes were incubated at room temperature for 10mins before administration to cells at 50nM (1.5nmol siRNA per plate containing 3mL antibiotic-free media). Cells were incubated with Lipofectamine-siRNA for 48 hours before collection for analysis. For complexation with JetPEI: in one vial, 1.5uL of siRNA (100μM in nuclease-free H_2_O) was diluted in 25μL of a 10% glucose solution (1:1 H_2_O/10% glucose, v/v, equivalent to a 5% glucose solution). In another vial, 0.24μL of JetPEI was diluted in 25μL of a 10% glucose solution and then added to the siRNA solution. The siRNA-JetPEI complex was incubated at room temperature for 10mins before administration to cells. Cells were incubated with JetPEI-siRNA for 24hrs or 48 hours before collection for analysis. Specific siRNA sequences are as follows: FoxM1 Sense, 5'-GGACCACUUUCCCUACUUUUU-3', FoxM1 Antisense, 5'-UUAAAGUAGGGAAAGUGGUCC-3', Control Sense, 5'-AACAGUCGCGUUUGCGACUGGUU-3' and Control Antisense, 5'-UUGUCAGCGCAAACGCUGACC-3'.

### Western Blot analysis of cell lysates

Cells were harvested with IP lysis buffer (20mM HEPES, 1% Triton X-100, 150mM NaCl, 1mM EDTA, 1mM EGTA, 100mM NaF, 10mM Na_4_P_2_O_7_, 1mM Na_3_VO_4_, 0.2mM PMSF). For analysis of tissues, liquid N_2_-frozen sections of xenograft tumors were homogenized in 1mL IP lysis buffer. Protein concentrations of cell or tumor lysates were measured by the Bio-Rad Protein Assay and protein separation was performed on 8% or 12% SDS-PAGE gels. Separated proteins were then transferred onto PVDF membranes (Millipore) and immunoblotted with specific antibodies against FOXM1 (c-20, Santa Cruz), Aurora B Kinase (cell signaling), cdc25B (cell signaling) and β-actin (Sigma)

### Quantitative RT-PCR of cell lysates

Monolayer cells and tumor sections were treated with Trizol for RNA isolation. For cells *in vitro*, media was removed from plates and cells were collected after addition of 1mL Trizol. For tumors, N_2_ (l)-frozen sections were homogenized (Fisher, Polytron) in 1mL Trizol. After treatment with Trizol, RNA was isolated by standard methods, comprising of chloroform extraction, precipitation with isopropanol, pellet washing with 75% EtOH (in H_2_O) and redissolving in nuclease-free H_2_O. cDNA was synthesized using the SuperScript First Strand Synthesis Kit according to the manufacturer's recommendations (Invitrogen). 2μg of cDNA was used with specific primers and SYBER-Green I for quantitative analysis of mRNA concentrations on an ABI 7900 HT system. Cyclophilin mRNA levels were used as normalization controls. Primer sequences are as follows: FoxM1 Sense- 5'- TCCCTGCTGCCTGATTATGC-3', FoxM1 Antisense- 5'- TCACCATTGCCTTTGTTGTTCC-3', Aurora B Kinase Sense- 5'- CTGGAATATGCACCACTTGGA-3', Aurora B Kinase Antisense- 5'- CGAATGACAGTAAGACAGGG-3', cdc25B Sense- 5'- CCCTTCCCTGTTTTCCTTTC-3', cdc25B Antisense- 5'- ACACACACTCCTGCCATAGG-3', Cyclophilin Sense- 5'- CACCCTGACACATAAACCCTGG-3', Cyclophilin Antisense- 5'- GCAGACAAGGTCCCAAAGACAG-3'.

### Animal maintenance and tumor xenograft experiments

Animals were maintained and treated in accordance with the guidelines established by the Animal Care and Use Committee of UIC. Tumor models were prepared by implanting cancer cell lines (1x10^6^ of MDA-MB-231-luc), suspended in 50uL of 1:1 PBS/Matrigel into each flank of 4-week old male athymic mice (Taconic). Treatment began once tumors reached sizes of 200mm^3^. After completion of the dosing schedule, animals were sacrificed and tumors were removed. Tumors were sliced in half and either frozen in liquid N_2_ or fixed overnight in 10% formalin (4°C). Frozen tumors were then sliced and homogenized in 1mL of either IP lysis buffer, or in Trizol, or western blot and RT-PCR analysis, respectively.

### Tumor-retention of fluorescent-siRNA

To study the retention of JetPEI-siRNA (AF488) in MDA-MB-231-luc xenograft tumors, 10μg Fluorescent-siRNA (encapsulated with 1.6μL JetPEI in 50uL 5% glucose solution by method described before) was injected directly into tumor tissues (50μL per tumor). Animals were anaesthetized under isofluorane and imaged for whole-body fluorescence (ex= 465nm, em=550nm) at 0hr, 12hr and 24hr post-administration using the Xenogen IVIS imaging system.

### Treatment of tumors with functional siRNA (anti-luciferase and anti-FoxM1)

Anti-luciferase and anti-FoxM1 siRNA were administered to MDA-MB-231-luc subcutaneous tumors (200mm^3^) at 10μg per tumor (complexed with 1.6μL JetPEI in 50μL of 5% glucose solution). For Luciferase experiments, tumors were injected with either anti-Luciferase siRNA or control siRNA, after which tumor-associated fluorescence was monitored by whole body bioluminescence imaging. To do so, animals were injected with 100mg luciferin/kg 10 mins before animals were anaesthetized under isoflurane. Tumor-associated bioluminescence were recorded on a Xenogen IVIS imaging system and quantified as flux (photon/sec) using the Living Image Software.

For functional suppression of FOXM1, anti-FoxM1 siRNA/JetPEI (10μg in 5μL 5% glucose solution) was administered to tumors 3 times a week for 3 weeks (total 10 injections, 50μL per injection). Control animals were treated with JetPEI only (1.6μL per tumor, 50uL per injection). After the treatment schedule, animals were euthanized and tumors removed and frozen in liquid N_2_. siRNA sequences for anti-FoxM1 and control siRNAs are stated above and those for anti-luciferase siRNA are as follows: Sense- 5'-r(CUUACGCUGAGUACUUCGA)d(TT)-3' and Antisense- 5'-r(UCGAAGUACUCAGCGUAAG)d(TT)-3'.

## CONCLUSIONS

In this report, we describe the effect of administering anti-FoxM1 siRNA into MDA-MB-231-luc breast cancer tumors. Firstly, we show that intratumoral injections of PEI-encapsulated siRNA was able to be retained within tumors, and secondly, we demonstrate its ability to induce a specific protein suppression effect. Furthermore, we show that the intratumoral injection of FoxM1-specific siRNA is able to suppress the protein expression of FOXM1, along with the protein and gene expressions of its transcriptional targets. These results showcase the effect of anti-FoxM1 siRNA in tumors, and highlight the potential of FOXM1-targeting as part of a cancer therapy.
